# MicroRNA-574 Impacts Granulosa Cell Estradiol Production *via* Targeting TIMP3 and ERK1/2 Signaling Pathway

**DOI:** 10.3389/fendo.2022.852127

**Published:** 2022-06-23

**Authors:** Bo Pan, Xiaoshu Zhan, Julang Li

**Affiliations:** ^1^Department of Animal Biosciences, University of Guelph, Guelph, ON, Canada; ^2^Cell and Developmental Biology Center, National Heart, Lung, and Blood Institute, National Institute of Health, Bethesda, MD, United States

**Keywords:** Ovary, Granulosa cell, microRNA, miR-574, Estradiol, ERK1/2 phosphorylation, TIMP3

## Abstract

Estradiol represents a key steroid ovarian hormone that not only plays a vital role in ovarian follicular development but also is associated with many other reproductive functions. Our primary study revealed that miR-574 expression decreased in porcine granulosa cells during development from small to large follicles, and the increase of ERK1/2 phosphorylation accompanies this change. Since it has been well established that the ERK1/2 activity is tightly associated with granulosa cell functions, including ovarian hormone production, we thus further investigate if the miRNA is involved in the regulation of estradiol production in granulosa cells. We found that overexpression of miR-574 decreased phosphorylated ERK1/2 without affecting the level of ERK1/2 protein, and on the other hand, the inhibition of miR-574 increased phosphorylated ERK1/2 level (P<0.05); meanwhile, overexpression of miR-574 increased estradiol production but knockdown of miR-574 decreased estradiol level in granulosa cells. To further identify the potential mechanism involved in the miR-574 regulatory effect, *in silico* screening was performed and revealed a potential binding site on the 3’UTR region of the tissue inhibitor of metalloproteinase 3 (TIMP3). Our gain-, loss- of function experiments, and luciferase reporter assay confirmed that TIMP3 is indeed the target of miR-574 in granulosa cell. Furthermore, the siRNA TIMP3 knockdown resulted in decreased phosphorylated ERK1/2, and an increase in estradiol production. In contrast, the addition of recombinant TIMP3 increased phosphorylated ERK1/2 level and decreased estradiol production. In summary, our results suggest that the miR-574-TIMP3-pERK1/2 cascade may be one of the pathways by which microRNAs regulate granulosa cell estradiol production.

## Introduction

Female reproductive success depends on ovarian follicular development which is reflected by the differentiation of granulosa cells and oocyte maturation. During the antral follicular developmental stage, somatic cells, especially granulosa cells, play an important role in oocyte competency acquisition (1, 2). In the ovary, estradiol is produced within the follicles, and is synthesized from androgen *via* aromatization by cytochrome P450 aromatase in granulosa cells (3). The significant role of estradiol in the ovary and other reproductive organs has been well documented. However, the underlying mechanisms in the regulation of its production within the follicles is still unclear.

MicroRNAs (miRNAs) are small, endogenous non-coding RNAs that can cause mRNA degradation or translation inhibition *via* imperfectly binding to its target mRNAs at their 3’ untranslated regions (3’UTRs). It was suggested that a single miRNA can potentially impact the expression of hundreds of mRNAs (4). Hence, miRNAs are related to diverse cellular processes and regarded as powerful and precise regulatory elements. Emerging evidence has shown that miRNAs are one of the critical regulators to ovarian functions (5, 6).

Due to its importance in ovarian function, the regulation of estradiol in granulosa cell have been an area of active study. Donadeu and his colleagues found not only miR-202 and miR-873 predominantly expressed in mural bovine granulosa cells, but also their expression is tightly correlated with estradiol levels and cytochrome P450 family 19 subfamilies A member 1 (CYP19A1) expression. Therefore, they suggested that miR-873 and miR-202 may be used as indicators of steroidogenic capacity in bovine (7, 8). In cultured mouse granulosa cells, miR-383 was one of the most significantly down-regulated miRNAs after TGF-β1-treatment. It was further found that miR-383 promoted estradiol production while its expression is under the suppression of steroidogenic factor-1 (SF-1) (9). Interestingly, the expression of SF-1 is down regulated by miR-764-3p, one of the most up-regulated miRNAs in TGF-β1-stimulated mouse ovarian granulosa cells (10). Our previous studies revealed that miR-378 regulates estradiol production *via* targeting the aromatase at the posttranscriptional level in both mural granulosa cells and cumulus granulosa cells during follicle maturation both *in vitro* and *in vivo* (11, 12). In addition, miR-574 was found to present in the ovarian cells and follicular fluid at a significant level (13), and its abnormal expression is associated with follicular atresia, Polycystic Ovary Syndrome (PCOS) and ovarian cancer (14–17). We have previous shown that miR-574 involved in regulating cumulus expansion and oocyte maturation *via* targeting hyaluronan synthase 2 (HAS2), a key enzyme in the formation of the hyaluronan (HA)-rich extracellular matrix during cumulus oocyte complex (COC) expansion (18). However, the role of miR-574 in regulating other ovarian functions, especially estradiol production is unknown. Therefore, the overall objective of the current study was to investigate if miR-574 regulates granulosa cell estradiol production, and if yes, what are the mechanisms behind the regulation.

Extracellular matrix (ECM) is a highly dynamic noncellular component that provides essential physical scaffolding for the cellular constituents and facilitates signaling required for tissue morphogenesis, differentiation and homeostasis. The tissue inhibitors of metalloproteinases (TIMPs) are known to directly restrict ECM proteolysis or indirectly promote ECM deposition (19). Four homologous members of the TIMP family have been identified and orthologs of the TIMPs are widely distributed across the animal kingdom (20). Among them, TIMP3 shows the broadest spectrum of inhibition of metalloproteases. It is a significant matrix protein localized to ECM through interaction with heparan sulfate and other sulfated proteoglycans (21, 22). Previous studies have shown that its mRNA and protein are abundantly expressed in human granulosa cells within the ovary across pre-, early, and late ovulatory follicular stages; and *TIMP3* mRNA expression increases in human granulosa cells from the early to the late ovulatory stage (23). Consistent with our previous research that TIMP3 is tightly associated with pig cumulus-oocyte-complex matrix remodelling during the *in vitro* cumulus cell expansion and oocyte maturation (24), the investigation in to the regulatory mechanisms controlling the expression of TIMP3 mRNA in periovulatory rat granulosa cells suggested it plays a unique role in the processes of ovulation and luteinization *via* regulating the fatty acid synthesis and steroidogenesis (25). However, whether the TIMP3 is involved in the regulation of estradiol production and if its expression is regulated by microRNAs in the ovary was unclear.

## Materials and Methods

Unless otherwise noted, all chemicals and reagents were purchased from Sigma-Aldrich Chemical Co. (St. Louis, MO). Molecular biological enzymes, molecular size markers, culture media, and Trizol were purchased from Invitrogen Life Technologies, Inc. (Carlsbad, CA). Oligonucleotide primers were ordered from the Integrated DNA Technologies, Inc. Recombinant TIMP-3 Protein (Cat #: 973-TM-010) was purchased from the R&D Systems company, and 0.1µg/ml was used for this study.

### Pig Granulosa Cell Isolation and Culture

**Figure 1:**
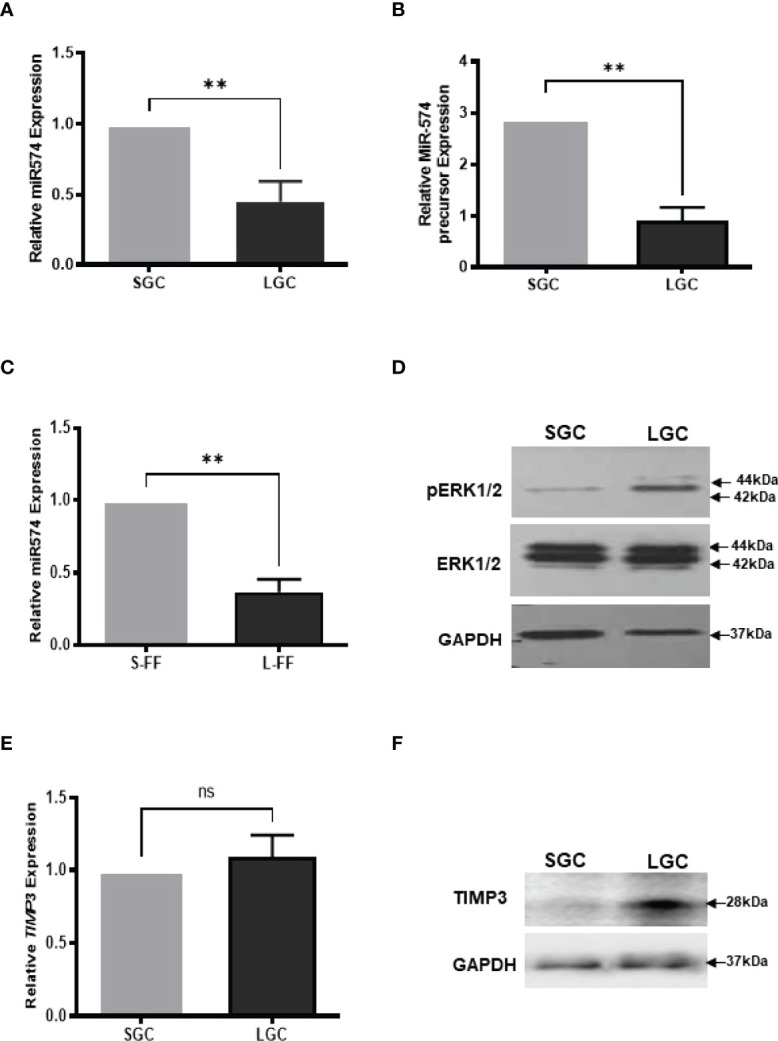
The expression of miR-574, pERK1/2 and TIMP3, during the antral follicular maturation. **(A)** The quantitative real-time PCR analysis of mature miR-574 in granulosa cells (GC) isolated from small-size (1-3 mm) and large-size (4-6 mm) follicles. **(B)** The quantitative real-time PCR analysis of miR-574 precursor in LGCs and SGCs. **(C)** The quantitative real-time PCR analysis of miR-574 in the follicular fluid (FF) isolated from small (S-FF) and large (L-FF) follicles. **(D)** Western blot analysis image showing increase pERK1/2 in LGC compared to SGC. **(E)** The quantitative real-time PCR analysis of *TIMP3* mRNA in granulosa cells (GC) isolated from small- and large follicles. **(F)** Western blot analysis image showing increase TIMP3 in LGC compared to SGC. ns, no significant difference. Error bars represent means ± SD of at least three biological replicates and Asterisks denote a significant difference between groups (P<0.05) as determined by Student’s *t*-test.

All animal procedures were performed in accordance with the guidelines established by and with the approval of the Animal Care Committee at the University of Guelph. The procedure of ovary collection, isolation and the culture of granulosa cells were described previously (26). Briefly, porcine ovaries were collected from prepubertal gilts at a local slaughterhouse, transported to the laboratory within two hours while maintained at room temperature, and rinsed three times with sterile 1X PBS. Granulosa cells were aspirated from small (SG; 0.5–3 mm in diameter) and large (LG; 4–6 mm in diameter) follicles using a 20-mL syringe fitted to an 18-gauge needle. Cells and follicular fluid were centrifuged at 500 × g for 5 min. Cells were washed with a large volume of DMEM/F12 (Gibco, Carlsbad, CA, USA) supplemented with 1X antibiotic/antimycotic (Gibco). Cells were dispersed by pipetting and washed two additional times. Viable cells, determined by trypan blue exclusion, were seeded at 0.6 × 10^6^/mL in 24-well tissue culture-treated plates in DMEM/F12 with 10% FBS (Gibco) and 1X antibiotic/antimycotic (Gibco). Cells were cultured in a humidified atmosphere of 5% CO_2_ at 38 °C. The medium was removed after 24 h, cells were washed with 1X PBS, and fresh DMEM/F12 with 10% FBS was added to continue the primary culture. With the exception of data in **Figure 1**, all the rest of the studies was performed using granulosa cells isolated from small follicles.

### Production of Recombinant MiR-574 Lentivirus and Viral Transduction

The lentiviral gene transfer plasmids pLV-[hsa-mir-574] (Cat.no. mir-p374) and pLV-[miR-control] (Cat.no. mir-p000) plasmid were purchased from BioSettia (San Diego, California, USA). Production of recombinant lentiviral particles was performed by following the manufacturer’s protocol using the HEK-293FT packaging cell line. The viral transduction of cultured granulosa cells was performed as previously described (11). Briefly, granulosa cells were isolated from pig ovaries and cultured overnight to reach 40–50% confluence. Then the cultured granulosa cells were transduced with lentivirus by replacing media with the appropriate volume of lentivirus-containing supernatant with 8 µg/mL polybrene (Sigma). Cells were incubated for 18h before the lentiviral media was replaced with fresh DMEM/F12 containing 10% FBS and 1X antibiotic/antimycotic (Gibco) and cultured for an additional 48 h. Cells were collected and stored at −80°C until analysis.

### Antisense Inhibition of miR-574 Expression

Specific anti-miR miRNA inhibitor of mir-574 (cat. no. 4464084; Life Technologies) and anti-miR miRNA inhibitor negative control (cat. no. AM17010; Life Technologies) were used to transfect culture pig granulosa cells according to the manufacturer’s instruction. Lipofectamine 3000 (cat. no. L3000001; Life Technologies) reagent was used for transfection with anti-miR-574 or negative control at final concentrations of 20 pmol/ml or 40 pmol/ml. The spent medium was collected at 24 h or 48 h after transfection and stored at 80°C until analysis.

### Transient Transfection and Luciferase Assay

Fresh granulosa cells were seeded and cultured in 24-well plates (Eppendorf, 0030722116), and the cell confluence usually reached 50 – 70% after 24 h culture. Transfection was performed with Lipofectamine 3000 (Invitrogen, L3000015) according to the manufacturer’s instruction (1:3 ratio of DNA/Lipofectamine). The Stratagene PathDetect Elk1 trans-reporting system (Agilent Technologies, Cat.219005) was used to determine the treatment indirectly/directly stimulates Elk1 or CREB through phosphorylation of ERK1/2, respectively. The Elk1 trans-reporting system package includes pFA2-Elk1 (activator plasmid), pFC2-dbd plasmid (negative control plasmid), and pFC-MEKK plasmid (positive control plasmid). Approximately 425 ng of GAL4-luciferase (luciferase reporter plasmid) and 25 ng pRL-TK (control reporter plasmid expressing Renilla luciferase) were co-transfected into the cultured GCs according to kit recommendations. Cells were cultured and grown in Dulbecco modified Eagle medium (DMEM, Invitrogen) supplemented with 10% fetal calf serum (FCS) in the first 24 h and in medium containing 0.5% FBS for the final 24 h. Then, the DNA complexes were removed by refreshing with the new medium. The cells were starved in DMEM/FCS-free for 20 h before used for pDEFB103A or vehicle treatment experiments. After 6 h incubations, cells were washed with ice-cold PBS and lysed with 100 μl Passive Lysis Buffer. Luciferase assays were performed by following the Dual-Luciferase Reporter Assay System instruction (Promega, Cat.E1960). Reporter activity was calculated as relative luciferase activity (Firefly luciferase/Renilla luciferase) to correct for transfection efficiency and each experiment was performed at least three times.

### RNA Isolation, Reverse Transcription and Real-Time Quantitative PCR(RT-qPCR)

Total RNA was isolated from fresh or cultured granulosa cell samples by using the Total RNA Purification Kit in accordance with the manufacturer’s instruction (Norgen Biotek, Thorold, Ontario, Canada). The RNaseFree DNase I Kit (Norgen Biotek) was applied to minimize amounts of genomic DNA contamination. RNA quality was measured by using a NanoDrop-8000 (Thermo Scientific, Waltham, MA) where the 260/280 ratio values are commonly 2 indicating “pure” RNA. In addition, RNA integrity was confirmed by gel electrophoresis devoid of genomic DNA contamination. For each sample, 500 ng of total RNA was used, and first-strand cDNA synthesis was carried out using the SuperScript II System according to the manufacturer’s instruction (Life Technologies, Inc.) and conducted in T100TM Thermal Cycler (Bio-Rad). The cDNA was diluted 1:20 for use in real-time PCR. Real-time PCR analysis was performed using the primers shown in **Table 1**. Real-time RT-PCR was performed on the Bio-Rad CFX ConnectTM Real-Time System (Bio-Rad) using the Sso-Advanced™ Universal SYBR Green Supermix (Bio-Rad). The PCR reaction consisted of 10 μl of SYBR Green PCR Master Mix, 100 nm of forward and reverse primers, and 2.0 μl of 1:20-diluted template cDNA in a total volume of 20 μl. Melting curve analyses were performed following the RT-qPCR, and the specificity of the PCR amplification products was confirmed by the presence of a single peak and the PCR product size was confirmed by gel electrophoresis with no visible primer-dimer products. All the primers were designed either to span an intron or to target exon–exon junctions to guarantee our cDNA template was free of contaminating gDNA. Primer efficiencies and PCR amplification efficiencies assay were generated using a serial dilution of cDNA templates with a range from 95% to 105%. Glyceraldehyde 3-phosphate dehydrogenase (GAPDH) was used as reference genes, and a geometric mean of their Ct value was used as an internal control to calculate the relative expression level of the target gene using the Ct method. All real-time PCRs were performed at least three times, and the changes in gene expression were reported as fold increases relative to untreated controls. For microRNA expression test and normalization, all the reagents, including miR-574 primer (Cat: 206011) and reference gene U6 snRNA primer (Cat: 203907), reverse transcription (Cat: 203301) and ExiLENT SYBR® Green master mix (Cat: 203403) were purchased from EXIQON (Exiqon, MA 01801, USA) and the reverse transcription and RT-qPCR for microRNA test were performed according to the manufacturer’s instruction.

**Table 1:** The primers used for Real-time qPCR.

GENES	SEQUENCE	PRODUCT	GENE NO
TIMP3	AGCGCAAGGGGCTGAACTATCG CGGGTAGCCGAAATTGGAGAGC	263	AF156031
GAPDH	GTTCCAGTATGATTCCACCCACGGCA TGCCAGCCCCAGCATCAAAGGTAGAA	147	NM_001206359.1
Pre-miR574	GTGTGGGTGTGTGCATGAG GCCTTGGGGGTGAAGGTC	75	NR_038571.1
Aromatase	GGGTCACAACAAGACAGGACT ACCTGGTATTGAAGATGTGTTTTT	202	NM_214429
Cyp450scc	TTCCAGAAGTATGGTCCCATTTA TGAGCATGGGGACACTAGTGTGG	255	NM_214427

### Western Blotting Analysis

Total proteins were isolated from granulosa cells. The lysates were obtained by lysing cells in Radioimmunoprecipitation assay buffer containing 1 mM of phenylmethylsulfonyl fluoride (Tribioscience Inc., Palo Alto, CA) and 1× protease inhibitor cocktail (Cedarlane Laboratories Limited, Hornby, Ontario, Canada). Lysed samples were mixed with 4× laemmli sample buffer (Cat.161-0747, BioRad), then boiled for 10 min, and centrifuged for 3 min at 12,000 × g. The equal amounts of supernatant containing the soluble protein were subjected to 10% SDS-PAGE gel. The proteins were transferred to polyvinylidene difluoride membranes (Millipore, Billerica, MA) by a Trans-BlotR TurboTM transfer system (Cat. 1704150, BioRad). Membranes were blocked in PBS with 5% skim milk powder at 4°C overnight and then incubated with the primary antibodies. The primary antibodies include rabbit polyclonal ERK2 (K-23) antibodies (Cat.SC153; Santa Cruz Biotechnology, Santa Cruz, CA), anti-phosphoERK (Thr202/Tyr204) antibodies (Cat.#9101; Cell Signaling Technology, Danvers, MA), TIMP3 (Catalog Number: orb11485; Biorbyt), followed by incubation with secondary antibody anti-rabbit IgG horseradish peroxidase (Cat.#7074; 1:2000; Cell Signaling Technology) for at least one hour under room temperature. For detection of glyceraldehyde-3-phosphate dehydrogenase (GAPDH, used as a protein loading control), the lower blot was incubated with primary antibody against GAPDH (1:10,000; Abcam, Cambridge, MA), followed by incubation with anti-mouse IgG HRP (1:10,000). Proteins were detected by the Clarity Western ECL substrate and imaged with the ChemiDoc XRS+ System (Bio-Rad, Hercules, CA, USA).

### Construction of Luciferase Reporter

*In silico* analysis using rna22 (https://cm.jefferson.edu/rna22/Interactive/) and RNAhybrid (https://bibiserv.cebitec.uni-bielefeld.de/rnahybrid) to find potential binding site for miR-574 in the metallopeptidase inhibitor 3 (TIMP3) 3’UTR (Genebank accession: AF156031). Sections of 3’UTR were cloned into the CMV-driven luciferase reporter pMIR-Report (Addgene, Cambridge, MA, USA; Addgene plasmid 22790). The sequence was amplified from the pig TIMP metallopeptidase inhibitor 3 (TIMP3) 3′UTR sequence (Genbank accession XM_003126073.5) using primers: UTR-F: 5′-TCCCACAAGATTTCCTCCTG-3′ and UTR-R:3′-AGGAAGGAGGAGGGAAACAA-5′. Site-directed mutagenesis was performed on reporter constructs using the overlap PCR method as previously described (27). Briefly, mutations were introduced into the putative miR-574 binding site with the putative miR-574 binding site in bold and mutated sequence underlined. Luciferase reporter gene assay was performed as previously described (27). Briefly, fresh granulosa cells were seeded into a 24-well and transduction by lentivirus was performed at 40–50% cell confluence. Refreshed the culture medium 24 h later, cells were then cotransfected with the firefly luciferase reporter constructs [pMIR-Report vectors, described earlier, or pGL3- Basic (promoter less luciferase vector; Promega] and pRL-TK, a vector encoding the renilla luciferase reporter gene. Transfections were carried out using the Lipofectamine 2000 reagent (Invitrogen) following the manufacturer’s protocol and included a total of 0.55 μg of DNA (0.5 μg of each firefly luciferase construct and 0.05 μg of renilla luciferase reporter plasmid). Cell lysates were harvested by direct lysis after 30 h of culture. Luciferase activity was measured in triplicate using the Dual-Luciferase Assay System (Promega). Firefly luciferase activity was normalized by renilla luciferase activity, and each result was presented as the fold relative to pGL3-Basic as described previously (28). Each experiment was performed at least in triplicate.

### ELISA Assay

Spent medium was collected and centrifuged at 500×g to remove cells or debris. An aliquot of each sample was assayed for the estradiol (E2) level using an Estradiol ELISA kit (Cat. EA 70; Oxford Biomedical Research, Oxford, MI) according to the manufacturer’s protocol. Briefly, the E2 ELISA is based on the principle of competitive binding between E2 in the test specimen and E2-HRP conjugate for a constant amount of rabbit anti-Estradiol. In the incubation, goat anti-rabbit IgG-coated wells are incubated with E2 standards, controls, Estradiol-HRP Conjugate Reagent and rabbit anti-Estradiol reagent at room temperature for 90 min. During the incubation, a fixed amount of HRP-labeled E2 competes with the endogenous E2 in the standard, sample, or quality control serum for a fixed number of binding sites of the specific E2 antibody. E2 peroxidase conjugate immunologically bound to the well progressively decreases as the concentration of E2 in the specimen increases. Unbound E2 peroxidase conjugate is then removed and the wells washed. Next, a solution of TMB Reagent is added and incubated at room temperature for 20 min, resulting in the development of blue colour. The colour development is stopped with the addition of stop solution, and the absorbance is measured spectrophotometrically at 450 nm. A standard curve is obtained by plotting the concentration of the standard versus the absorbance. At the same time, an empty well was used as blank control for E2 to assess the basal level in media. The coefficients of variation of the ELISA assay were below 15%. The sensitivity of the ELISA assay is 15 pg/ml. The levels of E2 in all the samples were above the detection sensitivity. Each experiment was carried out in triplicate.

### Statistical Analyses

Data represent the mean ± SE of at least three independent experiments. Statistical differences between all groups were determined using a one-way analysis of variance (ANOVA) followed by Tukey’s test for multiple comparisons. Differences between the two groups were determined using Student’s t-test. Results were considered significant at P < 0.05.

## Results

### MiR-574 Decreased in Granulosa Cell, and Follicular Fluid During the Antral Follicular Maturation While Phosphorylated ERK1/2 and TIMP3 Increased

We first investigate the expression of miR-574 in granulosa cells and follicular fluid isolation from small (1-3 mm in diameter, SGC) and large (4-6 mm in diameter, LGC) size antral follicle, respectively. As shown in **Figures 1A, B**, miR-574 and its precursor were detected in granulosa cell while miR-574 was solely detected in follicular fluid (**Figure 1C**); and its expression significantly decreased during follicular development. Previous studies also demonstrated that, as one of identified differentially expressed miRNAs in follicular fluid, miR-574 is not generally found in serum, suggesting that miR-574 resources from the cells near the ovarian follicle rather than serum (29). Interestingly, as shown in **Figures 1D, F** the levels of phosphorylated ERK1/ERK2 and TIMP3 protein in granulosa cells inversely mirrored that of miR-574 during antral follicular development (**Figure 1D**), although the TIMP3 mRNA expression is not altered significantly.

### MicroRNA-574 Regulates Phosphorylated ERK1/2 Level and Estradiol Production

**Figure 2:**
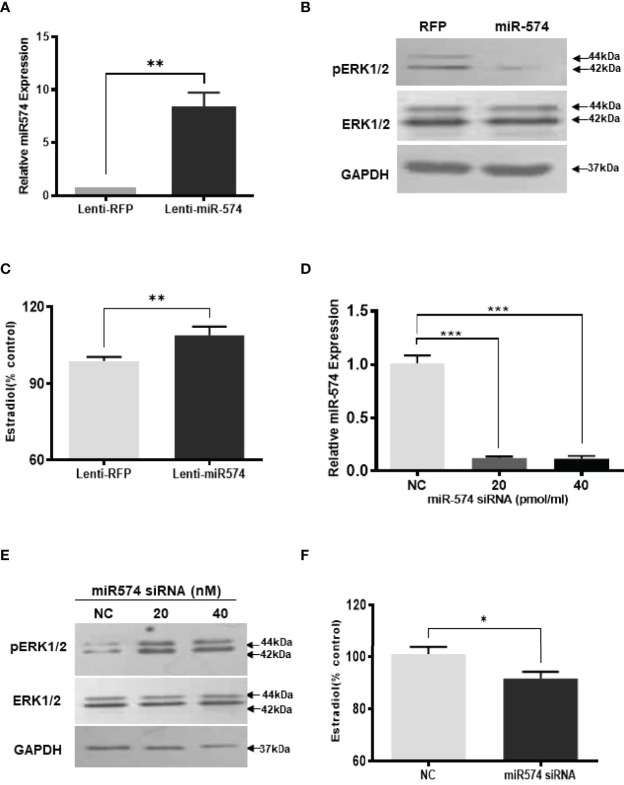
Gain- and loss-of-function of miR-574 change phosphorylated ERK1/2 level and Estradiol production in cultured granulosa cells. Cultured follicles granulosa cells were transduced with lentivirus that either expressed microRNA574 (miR574) or red fluorescent protein (RFP). **(A)** Relative miR-574 level after lentivirus transduction, snoU6 was used to normalize the mature miR-574 expression; **(B)** Representative western blot image of pERK1/2 and ERK1/2 protein in granulosa cells after overexpression of miR-574; **(C)** Estradiol production was measured by ELISA assay Overexpression of miR-574 promoted granulosa cells to produce more estradiol. **(D)** Relative levels of miR-574 after transfection with specific siRNA for miR-574 (miR-574 siRNA; 20 pmol/ml) and negative control siRNA (NC; 20 pmol/ml) in the pig granulosa cell. snoU6 was used to normalize the mature miR-574 expression data. **(E)** Representative western blot image of pERK1/2 and ERK1/2 protein in response to miR-574 downregulation. **(F)** Suppression of miR-574 resulted in granulosa cells to produce less estradiol. The data represents the mean ± SE of three independent experiments. Asterisks denote statistically significant differences between treatment and control groups (p <0.05).

To investigate if miR-574 may play a role on regulating the levels of phosphorylated ERK1/ERK2 in the granulosa cells, we generated miR-574 expressing (Lenti-miR-574) and control lentivirus (Lenti-RFP). As shown in **Figure 2A**, transduction of Lenti-miR-574 resulted in significant increases in miR-574 expression in the cultured granulosa cells compared to that of Lenti-RFP control group. To investigate whether miR-574 regulates pERK1/2 expression, we evaluated changes of ERK1/2 at protein levels by western blotting. No significant change of ERK1/2 was detected between the Lenti-RFP and Lenti-miR-574 groups while the phosphorylated ERK1/2 protein (pERK1/2) level was decreased in the Lenti-miR-574 group in granulosa cells (**Figure 2B**). As the production of estradiol in granulosa cell is under the control of the ERK pathway (30, 31), we next examined the influence of miR-574 on estradiol production. The spent media from the cultured Lenti-miR-574-transduced and Lenti-RFP-transduced cells were collected and ELISA was performed. As shown in **Figure 2C**, overexpression of miR-574 increased the production of estradiol in the granulosa cells.

If miR-574 suppressed pERK1/2 level and thus increased estradiol production in granulosa cells, one would expect the downregulation of miR-574 to reverse this effect. To verify this, inhibitors of miR-574, at 20 pmol/ml and 40 pmol/ml, were transfected to granulosa cells, they significantly decreased miR-574 when compared to the negative control which contained a random sequence with the same length as the specific inhibitor but does not target any known miRNA (**Figure 2D**). Down-regulation of miR-574 resulted in an increase of pERK1/2 without a change in the unphosphorylated ERK1/2 level (**Figure 2E**). Meanwhile, the inhibition of miR-574 also resulted in a decrease in estradiol production in the cultured granulosa cells (**Figure 2F**). Unexpectedly, the levels of estradiol in follicular fluid from large follicle were found to be not lower than that of the small follicle fluid (p>0.05; **Supplementary Figure 1**). It is possible that the estradiol production by granulosa cells accumulate follicular fluid during follicular development, and results in estradiol level in large follicles catching up with that of small follicles.

**Figure 3:**
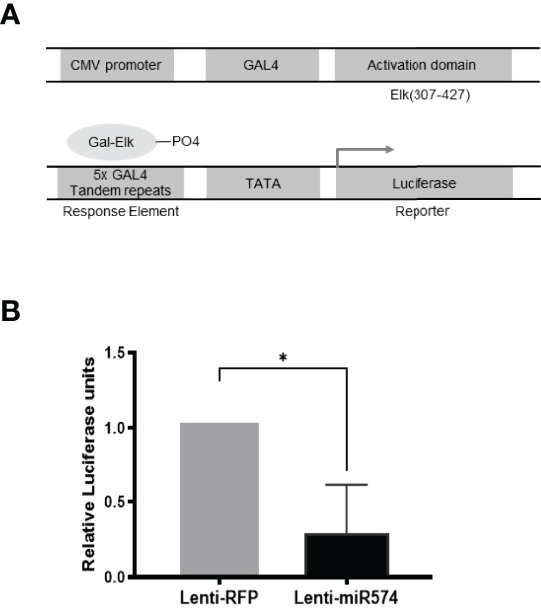
Over-expression of miR-574 decreases the activity of pERK1/2 indicated by the downregulation of pFA2-Elk1 luciferase activity. *in vivo* **(A)** The PathDetect *in vivo* signal transduction pathway trans-reporting systems was used to monitor the activation of ERK1/2 pathways. The PathDetect vectors containing a synthetic promoter with five tandem repeats of the GAL4 binding site, and pFA2-Elk1 that express the activation domain of Elk1 transcription factor fused to the GAL4 DNA binding domain. The activation of ERK1/2 kinases will result in the activation of its Elk trans-activators, which in turn stimulate reporter expression. **(B)** Luciferase activity, indicative of transcription factor dependent activation, is expressed as relative light units compared to control. The data represents the mean ± SE of four independent experiments. Asterisks denote statistically significant differences between the RFP and miR-574 group (p <0.05).

To further verify the role of miR-574 in the activation of the ERK1/2 pathway, we utilized the Agilent Technologies luciferase-based path-detect reporter system to monitor the activation of the transcription factor which is the downstream components of the ERK1/2. In this study, the activation of the ERK1/2 pathways by these constructs was monitored in parallel by co-transfections with luciferase-based Gal-Elk reporter systems. As shown in **Figure 3**, over-expression of miR-574 resulted in a decrease reporter (luciferase) activity which promoter harbors the Elk1 activation site. Taken together, these results suggest that miR-574 indeed suppresses ERK1/2 phosphorylation and activated ERK1/2 signalling pathway in the cultured granulosa cells.

### MiR-574 Modulates TIMP3 Expression in Cultured Granulosa Cells

**Figure 4:**
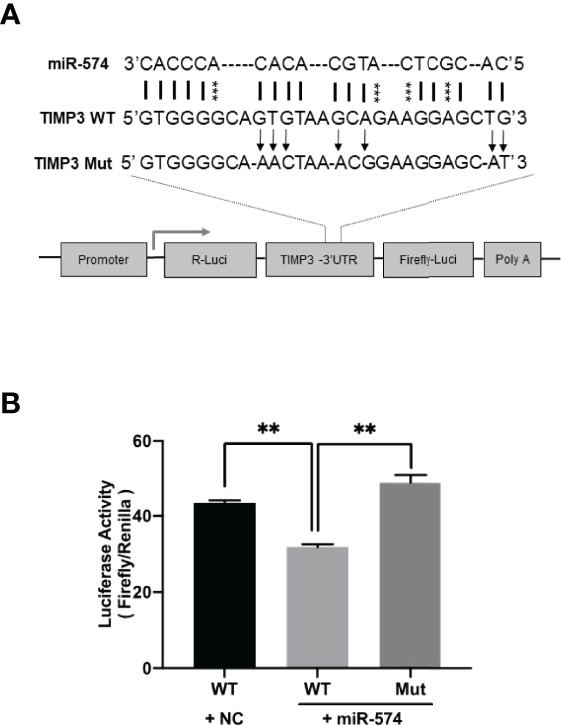
Identification of putative miR-574 binding sites within the TIMP3 3-UTR. **(A)** Putative miR-574 binding sites at the 3’UTR of TIMP3 were computationally identified by the online target detection program (https://bibiserv.cebitec.uni-bielefeld.de/rnahybrid); Top lines: miR-574 sequence; middle line: wild-type 3-UTR sequences; bottom line: mutated 3’UTR sequences. asterisks indicate the mutated nucleotides; **(B)** Granulosa cells were first transduced with lenti-miR-574 and then transfected with the luciferase-TIMP3 3-UTR vector or a control vector (a construct in which the 3-UTR of TIMP3 was replaced with a random sequence without any miR-574 target sites), and luciferase activity was measured. Mut represents luciferase expression constructs in which the miR-574 binding sites in the luciferase-TIMP3 3-UTR vector were mutated as indicated in **(A)**. The mean ± SEM of the relative luciferase expression ratio (Firefly luciferase/Rinella luciferase, Luc/R-luc) was calculated based on three biological replicates, and compared with the negative control (NC). Data represent the mean ± SE of three independent experiments. Asterisks denote statistically significant differences when compared to RFP (control; p <0.05).

Using the online RNAhybrid miRNA target detection program (Fast and effective prediction of microRNA/target duplexes RNA), the 3′-UTR of pig TIMP3 mRNA was predicted to have a putative target site for miR-574. To further verify if miR-574 indeed targets to the tentative site, a luciferase expression unit linking the WT and mutated 3’UTR of TIMP3 was constructed (**Figure 4A**) and transfected into granulosa cells. As shown in **Figure 4B**, in the presence of the wild type TIMP3 3’UTR, miR-574 mimic transfection resulted in decrease of luciferase activity when compared to control (randomized small RNA with no known target). This suppression of luciferase activity was absence when the miR-574 binding site in the TIMP3 3’UTR is mutated, suggesting miR-574 may regulate TIMP3 expression *via* targeting its 3’UTR.

**Figure 5:**
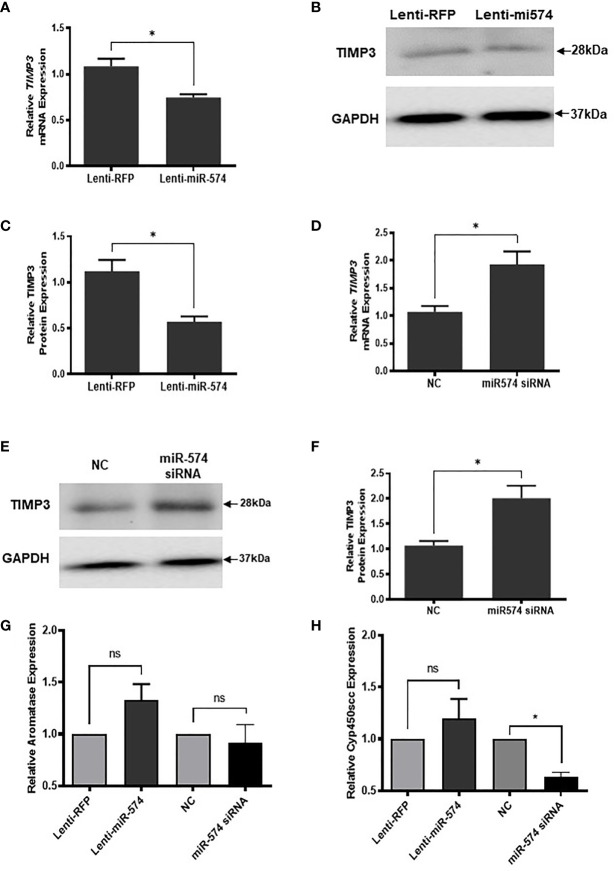
miR-574 regulates TIMP3 in the cultured granulosa cell. **(A)** Relative levels of *TIMP3* mRNA in the granulosa cell after overexpression of miR-574. **(B)** Representative western blot of TIMP3 protein in the granulosa cell after lentivirus transduction. **(C)** Densitometric quantitation depicting decreased expression of TIMP3 protein after over-expression of miR-574. GAPDH was used for western blot and mRNA normalization. The data represents the mean ± SE of three or four independent experiments. Asterisks denote statistically significant differences between the negative control (RFP) and miR-574 group (p <0.05). **(D)** Relative levels of *TIMP3* mRNA after transfection with specific siRNA for miR-574 (miR-574 siRNA) and negative control siRNA (NC) in the granulosa cells. **(E)** Representative western blot image of TIMP3 protein after siRNA of miR-574 transfection. **(F)** Densitometric quantitation depicting increased expression of TIMP3 protein. **(G)** Relative levels of Aromatase mRNA in the granulosa cell after overexpression or knock-down of miR-574. **(H)** Relative levels of Cyp450scc mRNA in the granulosa cell after overexpression and knock-down of miR-574. GAPDH mRNA and protein were used for western blot and real-time qPCR normalization, respectively. The data represents the mean ± SE of three independent experiments. Asterisks denote statistically significant differences between NC and miR-574-siRNA groups (p <0.05). NS, not significant.

To verify miR-574 indeed regulates TIMP3 expression in granulosa cell, the miRNA was overexpressed using the lentiviral transduction approach. **Figure 5** showed that both TIMP3 mRNA (**Figure 5A**) and protein (**Figures 5B, C**) were down-regulated when miR-574 was up-regulated. This finding was further confirmed by the loss of function studies. Transfection of siRNA against miR-574 resulted in increased TIMP3 expression at both mRNA (**Figure 5D**) and protein level (**Figures 5E, 5F**) in cultured granulosa cell. As showed in **Figures 5G, H**, while overexpression of miRNA resulted in a trend of increased *Aromatase* mRNA expression (p=0.1), it is not statistically significant. no change was observed in aromatase mRNA level in the miR-574 knockdown group. while si-miR-574 decreased the expression of this enzyme, no significant response was observed in *Cyp450scc* mRNA under miR-574 overexpression. These data suggest that the regulation of miR-574 on expression of these enzymes is not the major pathway for its increase on estradiol production.

### TIMP3 Impacts Estradiol Production *via* Regulating the Phosphorylated ERK1/2 Level in Cultured Granulosa Cells

**Figure 6:**
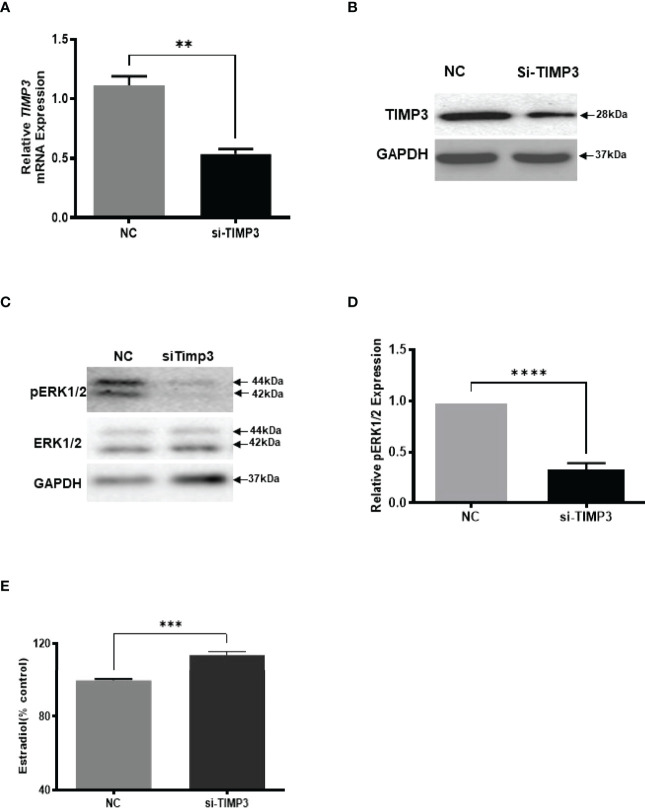
Inhibition of TIMP3 decreased phosphorylated ERK1/2 but increased estradiol production in cultured granulosa cells. **(A)** Relative levels of *TIMP3* mRNA after transfection with siRNA for TIMP3 (si-TIMP3) and negative control siRNA (NC) in granulosa cells. **(B)** Representative western blot of TIMP3 in granulosa cells after transfection with siRNA for TIMP3 (si-TIMP3) and negative control siRNA (NC). **(C)** Representative western blot of pERK1/2 and ERK1/2 protein after siRNA transfection. **(D)** Densitometric quantitation depicting decreased level of pERK1/2. **(E)** Suppression of TIMP3 promoted granulosa cells to produce more estradiol. GAPDH protein and mRNA were used for western blot data and mRNA normalization. Estradiol production was measured by ELISA assay. Data represent the mean ± SE of three independent experiments. Asterisks denote statistically significant differences between NC and si-TIMP3 group (p <0.05).

**Figure 7:**
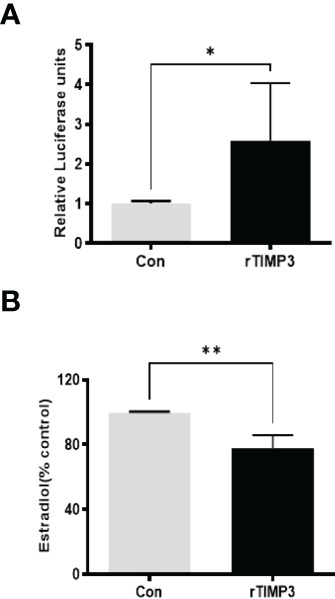
Recombinant TIMP3 increased phosphorylated ERK1/2 level and decreased estradiol production. **(A)** The PathDetect *in vivo* signal transduction pathway trans-reporting systems (Promega) was used to measure transcription activator and ERK1/2 signal transduction pathway after the addition of Recombinant TIMP3. First, the cultured granulosa cells were co-transfected with a pFR-luciferase reporter with a pFA2-Elk1 plasmid. 24h after the transfection, the recombinant TIMP3(rTIMP3) was added into the culture plate well. Granulosa cell sample was collected after 6h treatment then used for luciferase assay. Luciferase activity is indicative of transcription factor-dependent activation, is expressed as relative light units compared to control. **(B)** Estradiol production was measured by ELISA assay. The addition of TIMP3 suppresses estradiol production in the cultured granulosa cells. The data represents the mean ± SE of three or four independent experiments. Asterisks denote statistically significant differences between the blank control and the rTIMP3 group (p <0.05).

Given our finding that miR-574 regulated of ERK1/2 phosphorylation and its targeted relationship with TIMP3, we next asked if TIMP3 plays a role in the regulation of pERK1/2 level and estradiol production by the miRNA. SiRNA against TIMP3 was used to knock down TIMP3 expression in the cultured granulosa cells, both phosphorylated ERK1/2 and non-phosphorylated ERK1/2 were measured. As shown in **Figures 6A, B**, TIMP3 siRNA transfection resulted in a ~fifty percent knockdown of TIMP3, which is accompanied by a significant decrease of pERK1/2 level (**Figure 6C, D**). In addition, spent media was collected, and ELISA assay was performed to measure the estradiol concentration. As shown in **Figure 6E**, the repression of TIMP3 resulted in an increase of estradiol production in granuloma cells. To further verify the role of TIMP3 in estradiol production, recombinant TIMP3 was added to the culture media. The activation of the ERK1/2 pathways was monitored in parallel by co-transfections with luciferase-based Gal-Elk reporter systems. As shown in **Figure 7A**, the addition of recombinant TIMP3 (rTIMP3) resulted in an increase of Elk1-pathway reporter activity. Moreover, addition of rTIMP3 decreased the concentrations of estradiol in the granulosa cells (**Figure 7B**). The data from the gain- and lost- of function studies verifies the role of TIMP3 in the regulation of granulosa cell estradiol production.

## Discussion

The present study aimed to study the role of miR-574 in the regulation of estradiol production in porcine ovarian granulosa cells. Our study revealed novel layers in the regulation of this important process. First, miR-574 decreased pERK1/2, and increased estradiol production; second, miR-574 suppressed TIMP3 expression *via* targeting to its 3’UTR; and third, TIMP3 stimulated pERK1/2 and decreased estradiol production. Thus, our study collectively suggest a miR-574 -TIMP3- pERK1/2- estradiol pathway for the regulation of steroid hormone production in ovarian granulosa cells.

In the past two decades, much attention has been paid to the intracellular signalling molecules, the mitogen-activated protein kinase (MAPK) molecules, ERK1 and ERK2 in the regulation of ovary functions. It was well established that ERK1/2 serves as one of the intracellular signalling molecules in controlling granulosa cell estradiol synthesis, oocyte resumption of meiosis, ovulation and luteinization, in response to luteinizing hormone (LH) and follicle-stimulating hormone (FSH) (30, 32, 33). Within cells, the phosphorylation and dephosphorylation state of ERK1/2 represents the activation and inactivation of the ERK1/2 signalling pathway and tightly associated with critical intracellular and extracellular events. Comparing the phosphorylation level of ERK1/2 in the fresh isolated granulosa cells derived from small size follicles and large follicles, we observed that a high level of phosphorylated ERK1/2 was observed in the large follicular granulosa cells compared to the small follicular granulosa cells. This finding is consistent with previous report that ERK1/2 as the intracellular signaling molecules that differentially regulate FSH-induced hormone synthesis including progesterone and estradiol in rat granulosa cells (30).

A previous study investigating the potential role of TIMP3 in estradiol production in mammary gland tissue during the estrous cycle also revealed an inverted relationship between TIMP3 and estradiol level (34). Moreover, mice that lack TIMP1 exhibit a disruption of the estrous cycle length and increased estradiol level (35). Our finding is consistent with these reports on the role of TIMP3 in estradiol production regulation. We report alternation of TIMP3 level resulted in change of pERK1/2 level. TIMP3 is a matrix protein localized to ECM through interaction with heparan sulfate and other sulfated proteoglycans (21, 22), it is thus unlikely for TIMP3 to interact with ERK1/2 to increase its phosphorylation. The exact pathway on how TIMP3 trigger increase of ERK1/2 phosphorylation is currently unknown and worth further investigation.

Our finding that miR-574 levels in granulosa cells and in follicular fluid inversely mirrored the pERK1/2 level suggests that they may be correlated. The differential expression of miR-574 in the follicular fluid is suggested to associated with the *in vitro* fertilization outcome and ovarian ageing and dysfunction such as Polycystic Ovary Syndrome (13, 14, 36, 37). Thus, the observation of a significant decrease in miR-574 between the early antral follicular stage and later antral stage suggests it may play a role in fine tuning follicular development and maturation. It is well known that ERK kinases elicit biological outputs by phosphorylating nuclear and cytoplasmic substrates. A nuclear substrate such as transcription factors Elk-1 can be cloned into a luciferase plasmid vector under the control of a synthetic promoter, and transient transfection assay that monitors *in vivo* activation and translocation of ERK from the cytoplasm to the nucleus *via* measuring the luciferase activity (38). The confirmation of the activation of protein kinases ERK1/2 by miR-574, using the PathDetect signal transduction pathway trans-reporting systems further suggest the regulatory role of the miRNA to the activation of the pathway.

We next aimed to identify the potential miR-574 target in this regulation process. Owning to the fact the microRNAs play an essential role in gene expression regulation at post-transcriptional levels, and the observation of decrease miR-574 in the large follicle-derived granulosa cells. In an attempt to identify miR-574 target genes, we selectively focused the genes that were previously reported to be increased in the ovarian granulosa cells during growth from small to large antral sizes (39–41). By inputting the sequence of the 3’-UTR of these gene of interest *via* online and local target predicting tools, TIMP3 was identified as the best match. Interestingly, previous studies have shown that *TIMP3* mRNA is more abundant in healthy follicles, and an increase in its expression in granulosa cells from the early to the late ovulatory stage was observed (23, 42, 43). Our finding that *TIMP3* mRNA and protein increase in granulosa cells from small to the large follicle is consistent with the notion that a rise in *TIMP3* is required for the final stages of follicular maturation in preparation for the matrix remodelling in many species (23). We have previously reported that TIMP3 in the cumulus granulosa cell is tightly associated with pig cumulus-oocyte-complex matrix remodelling during the *in vitro* cumulus cell expansion and oocyte maturation (24). Our current findings show TIMP3 affected ERK1/2 and regulated estradiol production in granulosa cells, expanding our understanding of the role of this metalloproteinase in the ovarian follicle.

## Data Availability Statement

The raw data supporting the conclusions of this article will be made available by the authors, without undue reservation.

## Ethics Statement

The animal study was reviewed and approved by the Animal Care Committee at the University of Guelph.

## Author Contributions

All authors listed have made a substantial, direct, and intellectual contribution to the work and approved it for publication.

## Conflict of Interest

The authors declare that the research was conducted in the absence of any commercial or financial relationships that could be construed as a potential conflict of interest.

## Publisher’s Note

All claims expressed in this article are solely those of the authors and do not necessarily represent those of their affiliated organizations, or those of the publisher, the editors and the reviewers. Any product that may be evaluated in this article, or claim that may be made by its manufacturer, is not guaranteed or endorsed by the publisher.

## Supplementary Material

Supplementary Figure 1 Levels of estradiol in follicle fluid between small-size and large-size follicles. Follicular-fluid concentrations of estradiol were determined using ELISA assay kit. The pools of follicular fluid was aspirated from small (SG; 0.5–3 mm in diameter) and large (LG; 4–6 mm in diameter) follicles. Then centrifuged at 3000 g for 15 min to collect the supernatant. Serial dilutions of a pool of follicular fluid was performed to optimize the working concentration with ELLSA kit. A working dilution of 1:100 was used for the experimental follicular fluid samples. The data represents the mean ± SE of three or four independent experiments. Asterisks denote statistically significant differences between the blank control and the rTIMP3 group (p <0.05).
