# Supplementary figures and images for: MicroRNA-574 Impacts Granulosa Cell Estradiol Production via Targeting TIMP3 and ERK1/2 Signaling Pathway
Source: Front Endocrinol (Lausanne). 2022 Jun 23;13:852127. doi: 10.3389/fendo.2022.852127 (PMC9261285; doi:10.3389/fendo.2022.852127)

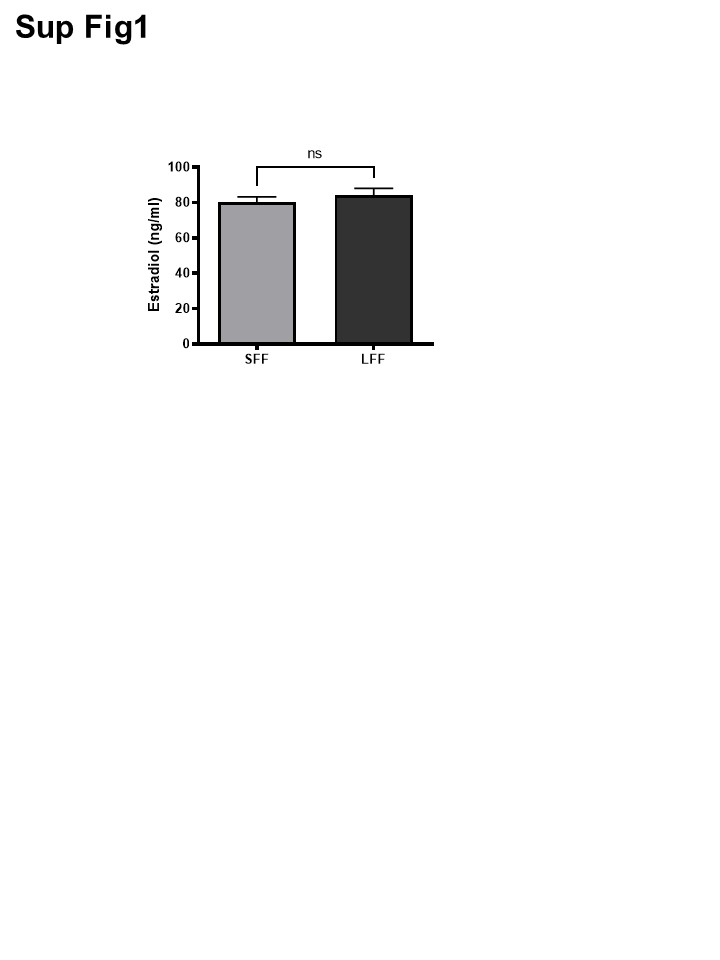

Supplement: Supplementary Figure 1 — Levels of estradiol in follicle fluid between small-size and large-size follicles. Follicular-fluid concentrations of estradiol were determined using ELISA assay kit. The pools of follicular fluid was aspirated from small (SG; 0.5–3 mm in diameter) and large (LG; 4–6 mm in diameter) follicles. Then centrifuged at 3000 g for 15 min to collect the supernatant. Serial dilutions of a pool of follicular fluid was performed to optimize the working concentration with ELLSA kit. A working dilution of 1:100 was used for the experimental follicular fluid samples. The data represents the mean ± SE of three or four independent experiments. Asterisks denote statistically significant differences between the blank control and the rTIMP3 group (p <0.05). [file Image_1.jpeg]
